# Towards mission-driven investment in new antimicrobials? What role for Chinese strategic industrial financing vehicles in responding to the challenge of antimicrobial resistance?

**DOI:** 10.1186/s12992-024-01030-2

**Published:** 2024-03-26

**Authors:** Lewis Husain, Yajing Hu, Yangmu Huang

**Affiliations:** 1 Institute of Development Studies, Brighton, UK; 2 Independent consultant, Beijing, China; 3 School of Public Health, Peking University, Beijing, China

**Keywords:** Global health, Antimicrobial resistance, Antibiotic resistance, Research and development, R&D, China, Industrial policy, Mission-driven innovation

## Abstract

**Background:**

Antimicrobial resistance (AMR) causes high levels of global mortality. There is a global need to develop new antimicrobials to replace those whose efficacy is being eroded, but limited incentive for companies to engage in R&D, and a limited pipeline of new drugs. There is a recognised need for policies in the form of ‘push’ and ‘pull’ incentives to support this R&D. This article discusses China, a country with a rapidly emerging pharmaceuticals and biotech (P&B) sector, and a history of using coordinated innovation and industrial policy for strategic and developmental ends. We investigate the extent to which ‘government guidance funds’ (GGFs), strategic industrial financing vehicles (a ‘push’ mechanism), support the development of antimicrobials as part of China’s ‘mission-driven’ approach to innovation and industrial policy. GGFs are potentially globally significant, having raised approximately US$ 872 billion to 2020.

**Results:**

GGFs have a substantial role in P&B, but almost no role in developing new antimicrobials, despite this being a priority in the country’s AMR National Action Plan. There are multiple constraints on GGFs’ ability to function as part of a mission-driven approach to innovation at present, linked to their business model and the absence of standard markets for antimicrobials (or other effective ‘pull’ mechanisms), their unclear ‘social’ mandate, and limited technical capacity. However, GGFs are highly responsive to changing policy demands and can be used strategically by government in response to changing needs.

**Conclusions:**

Despite the very limited role of GGFs in developing new antimicrobials, their responsiveness to policy means they are likely to play a larger role as P&B becomes an increasingly important component of China’s innovation and industrial strategy. However, for GGFs to effectively play that role, there is a need for reforms to their governance model, an increase in technical and managerial capacity, and supporting (‘pull’) incentives, particularly for pharmaceuticals such as antimicrobials for which there is strong social need, but a limited market. Given GGFs' scale and strategic importance, they deserve further research as China’s P&B sector becomes increasingly globally important, and as the Chinese government commits to a larger role in global health.

**Supplementary Information:**

The online version contains supplementary material available at 10.1186/s12992-024-01030-2.

## Introduction

Antimicrobial resistance (AMR) causes global mortality comparable to HIV/AIDS and malaria combined [1], and its burden of mortality and morbidity is expected to increase in the coming decades. Its economic impact by 2050 has been estimated at USD 100 trillion [2]. While many countries have implemented antimicrobial stewardship measures [3], there remains insufficient attention to developing the novel antimicrobials that are needed as older drugs lose their effectiveness [2, 4]. R&D for new antimicrobials has been declining since the 1980s [5], with only five new classes of antimicrobials brought to market since the beginning of the 21st century [6], and only 43 new drugs in clinical trials in December 2020, most of which fall within existing classes and are vulnerable to the rapid emergence of resistance [7].

There is an acknowledged need for public sector involvement in the development of new antimicrobials, to overcome market failure and provide drugs that are socially necessary, but which are insufficiently supplied by current market-driven arrangements. This ‘public goods’ nature of antimicrobials is summarised by O’Neill [2] as “things that benefit a wide group of people, where that group does not directly pay for their production”. Many procedures and much of the medical industry relies on the existence of functioning antimicrobials, but pharmaceutical companies have limited incentive to invest in their development, given the expense, risk, and likely limited market as new drugs must be carefully stewarded to slow the development of resistance. This usage of ‘public good’ is common in global health and development and differs slightly from an economic definition as it does not specifically articulate whether a given good is rival or excludable [8], and should perhaps be more accurately characterised as a ‘merit good’ [9]. In common with usage by O’Neill and others in this field, however, we use the term ‘public good’.

This has led to the development of a number of initiatives intended to incentivise basic research or to support companies to bring new products to market. Such initiatives are frequently characterised as either ‘push’ or ‘pull’ incentives. Push incentives include, for example, public investment in research, clinical trials, and support to companies to gain regulatory approvals. ‘Pull’ incentives, meanwhile, are intended to create incentives for companies by, for example, paying pharmaceutical companies for access to a drug, rather than paying for consumption by volume. Well-known examples of ‘push’ schemes include CARB-X and the Joint Programming Initiative on Antimicrobial Resistance (JPIAMR). Novel pull mechanisms include ‘access’ schemes, such as the United Kingdom’s ‘subscription’ model, being piloted by the National Institute of Health and Care Excellence [10].

More broadly, the experience of the COVID-19 pandemic, and the state-supported rapid development and roll out of vaccines, has strengthened calls for an increased public sector involvement in research and development (R&D) and ‘mission-driven’ industrial policy for addressing public health threats [11, 12].

There is a strong rationale for antimicrobial R&D efforts to be aligned globally, given the global nature of this challenge and the *global* public good character of novel antimicrobials [13]. WHO carries out assessments of the pipeline of new antibiotics under development and R&D priorities, based on clinical need [14]. However, many of the most prominent initiatives for promoting R&D for new antimicrobials are linked to high-income countries [13] and less is known about the place of antibiotics in the R&D priorities and industrial strategies of new, and emerging, biotechnology and pharmaceutical powers.

The article discusses the case of China, a country with rapidly increasing investment in biotechnology and pharmaceuticals [15] and a commitment to developing new antimicrobials. China also has a history of state involvement in industrial development and in shaping R&D around key national priorities, prompting questions around the extent to which the Chinese state has policy instruments that enable it to play a role in responding to the challenge of delivering public goods for health in a way that market economies may find harder. China’s engagement in the provision of COVID-19 vaccines demonstrated the country’s potential significance as a supplier of medical goods, while important international agencies including the Bill and Melinda Gates Foundation, the Coalition for Epidemic Preparedness, and PATH, all work in China to attempt to enhance the contribution of Chinese-developed health technologies to low-income country markets and global health.

The article focuses on Chinese ‘government guidance funds’ (GGFs, 政府引导基金), a key strategic industrial development instrument, and the extent to which they support companies developing antimicrobials or bringing them to market. As discussed below, the scale of funding through GGFs is very large, and they have been instrumental in the development of sectors of the Chinese economy such as microchips, which are of global significance. Through primary and secondary data analysis and interviews with GGF managers, it also explores the broader question of whether these funds have a potential role in supporting the development of health products with a ‘public goods’ character and in line with calls for increasing calls for ‘mission-driven’ approaches to innovation in the public interest.

The remainder of the paper is set out as follows: Sect. 2 discusses provides background on why Chinese GGFs are a relevant object of study in research on antimicrobial development in China. Section 3 outlines the methodology. Section 4 maps GGFs’ investments in P&B. Section 5 presents findings from key informant interviews to understand GGFs’ potential role in supporting the development of health products with a ‘public goods’ character. Section 6 presents a discussion of the findings in terms of theories of mission-driven innovation. Section 7 concludes.

## Background: why China? Why GGFS?

China is relevant to debates on global R&D for new antimicrobials for several reasons. First, the country has a substantial burden of AMR (e.g. [16]). and a long history of policy attention to this issue [17]. Second, the government committed in its 2016 National Action Plan (NAP) to developing 1–2 novel antimicrobials [18], and increasingly uses the language of ‘public goods’ to frame its role in global health [19]. Third, it has dramatically increased investment in its biotech and pharmaceutical industries, considered strategic pillar industries, and has a history of using coordinated policies and tools to support the development of key industries.

Since the start of economic reforms in the 1970s, the Chinese state’s role in the economy has been transformed. Government no longer directly manages the economy, and increasingly acts as a regulator, adjusting macro-economic levers to guide development [20]. However, the state retains tools to steer the economy for strategic ends, through development and industrial planning, which helps coordinate policies and channel resources towards key goals [21]. This includes in science and technology (S&T), which are central to plans for a transition to a higher-value, innovation-driven economy [22–24].

The Chinese government has used a range of coordinated push and pull mechanisms to develop the pharmaceuticals and biotechnology (P&B) sector. Push mechanisms include strategic research programmes to build systems and capacities for technology development [25], alongside pull mechanisms to strengthen regulation and promote market upgrading, and government purchasing to incentivise companies to innovate and upgrade [26, 27]. As well as focusing on leading-edge technologies, China’s strategy has focused on products relevant to the current stage of China’s development and society, for example R&D on communicable diseases such as TB [28], that continue to be a burden for China, and low-cost pharmaceuticals targeting low-income consumers. Such measures appear to be paying off: the country is now seen as an emerging biotech power [29], though sceptics caution that China does not yet have a globally competitive pharma industry [25, 30].

**Fig. 1:**
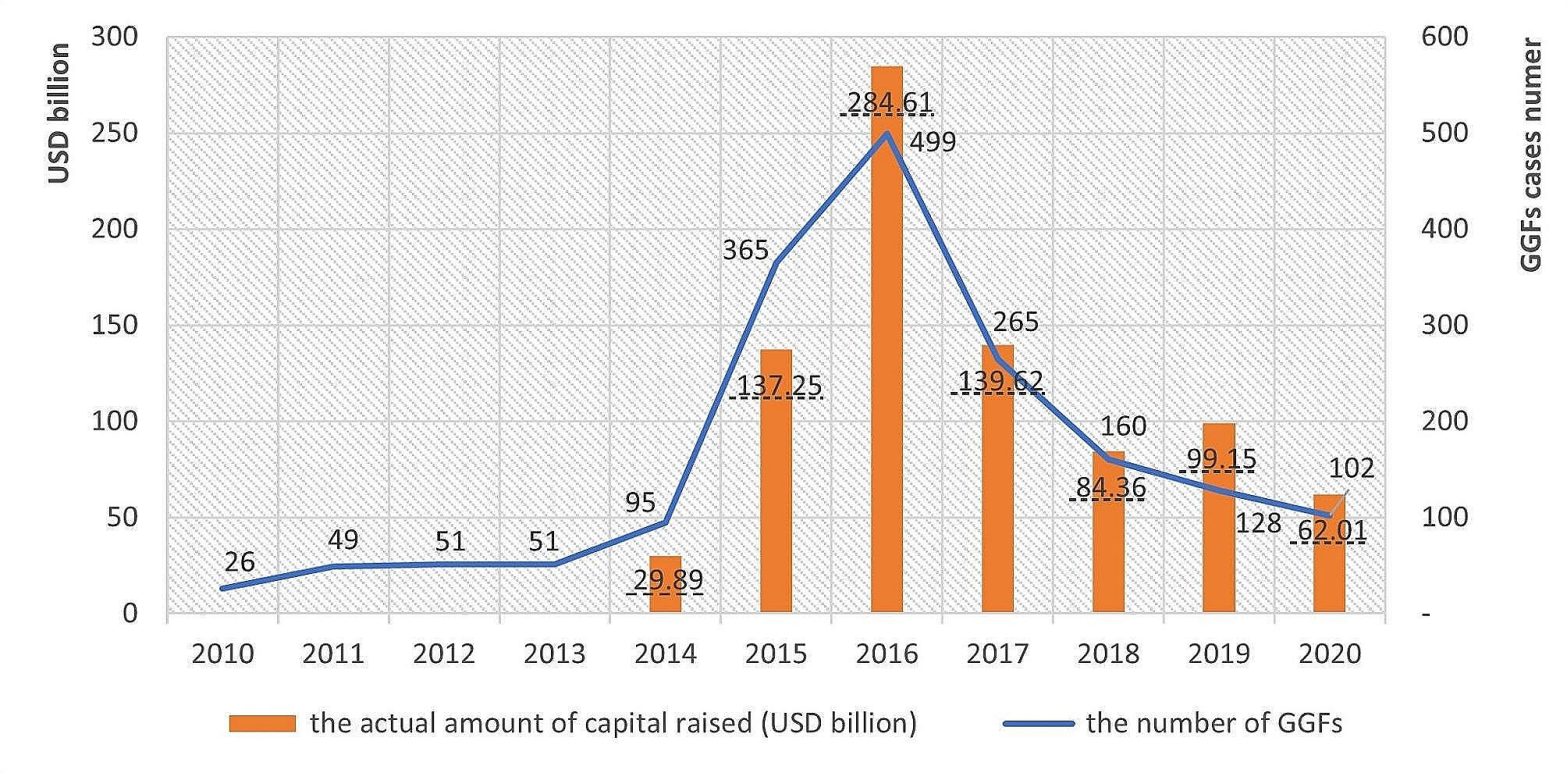
GGFs’ development and the capital they raised from 2010 to 2020. Source: [40]

GGFs are an important industrial development mechanism used across many sectors of the economy, including in P&B. GGFs are venture capital (VC) funds, used to channel investment to priority sectors and firms in line with the country’s strategic and developmental priorities [31, 32]. They are government-backed, and draw on government capital, while crowding in private capital [33]. They resemble government-backed VC funds elsewhere used to support innovation and start-ups [34] and are run by investment professionals, who make decisions about funding of individual projects or companies [35–37]. Although the first GGF was founded in 2002, GGFs really started to take off around 2014, coinciding with a renewed policy emphasis on innovation as a driver of growth in the Chinese economy [38, 39]. By the end of 2020, 1,851 GGFs had raised a total capital of USD 871.92 billion, making them a potentially globally very significant funding vehicle [40] (see Fig. 1). For reference, a detailed policy timeline is given in Additional file 2.

There has been little research on GGFs in English, but more in Chinese. English-language publications have tended to focus on GGFs’ role in reasserting state influence in the economy [19, 41]. In contrast, much Chinese-language analysis has discussed how GGFs support innovation, including through leveraging private investment in SMEs and increasing overall investment and business-level innovation [42, 43]. Government investment through GGFs can also help meet investment needs where the market fails to support innovation [44].

Other research shows how GGFs signal government’s policy preferences, influencing investment preferences and resource allocation [45, 46], increasing investors’ confidence [47], and guiding market development [48, 49]. This effect may extend to increasing innovative companies’ access to finance through the formal banking system [50], and crowd in certain preferential government policies [45].

Alongside GGFs’ role specifically in supporting companies developing antimicrobials, we are interested in the broader question of their potential role in supporting ‘mission-driven’ R&D in P&B, given China’s increasing global importance in this sector and its huge manufacturing capacity. ‘Mission-driven’ innovation has received increased attention in recent years, in the context of calls for strengthened state-led responses to global challenges through joined up industrial and innovation policy. Mazzucato argues that missions should demonstrate ‘directionality’ (setting the direction for problems to be solved), employ joined-up policy making, and involve different sectors and different types of policy actors [51], creating “a long-term public agenda for innovation policies, address[ing] societal demand or need, and draw[ing] on the high potential of the country’s science and technology system” [12]. Both supply- and demand-side policies, such as market shaping/creation, are important [52]. Mission-driven innovation is recognised as necessary to solving public health problems [14].

The scale of GGFs’ activity in the economy, and their ‘government-guided’ character make them significant in understanding the ways in which industries are being supported for strategic ends, including priorities associated with China’s current level of development, specific disease burden, or competition in key emerging technologies. To date, there have been very few sector-focused analyses of GGFs’ role (cf. [53])., and none focused on P&B, or key technologies such as antimicrobials, despite some recent research on the limitations of GGFs [54]. While we know of one working paper that discusses mission-driven innovation in the context of antimicrobials [55], no paper has looked at GGFs in this context. To our knowledge, none has looked at the extent to which GGFs are used in supporting the development of products with limited markets, such as antimicrobials, within the context of a strategic or developmental need.

## Methodology

The research employs data from PEDATA (leading commercial database of company information and investment trends) and companies’ websites to map GGFs’ investments, and interviews with GGF executives and managers.^1^ First, we collected data on GGFs investments in P&B from the earliest available data in PEDATA (2010) to August 2021. Data were extracted from two sub-databases of PEDATA, covering Biotechnology and Pharmaceuticals and Government Guidance Funds, allowing us to identify GGFs with investments in P&B. PEDATA does not include full details of all investments, so we accessed data on investments via company websites, supplemented with online searches. We then carried out interviews to obtain an in-depth understanding of GGFs’ operations and management. We interviewed 18 managers (see Additional File 1) from different GGFs across China. Interviewees were initially recruited through connections of one of the authors, with a background in Chinese equity investment, supplemented with snowballing. All interviewees were highly experienced in fund investment, in both the public and the private sectors. Interviews were recorded or notes were taken, depending on the preference of the interviewee, and transcripts/notes were coded manually by two of the authors. The primary purpose of the interviews was to understand how GGFs make decisions regarding potential investments and the extent to which they can contribute to the development of public goods, such as antibiotics. We summarise our interviewees’ answers in the following sections and quote specific responses where this helps illustrate certain points.

## Results: GGFS’ investments and potential to contribute to mission-driven innovation in P&B

### GGFs’ investments in P&B

This section describes GGFs’ investment in P&B, and factors influencing where, when and how they invest.

#### What do market data tell us?

**Fig. 2:**
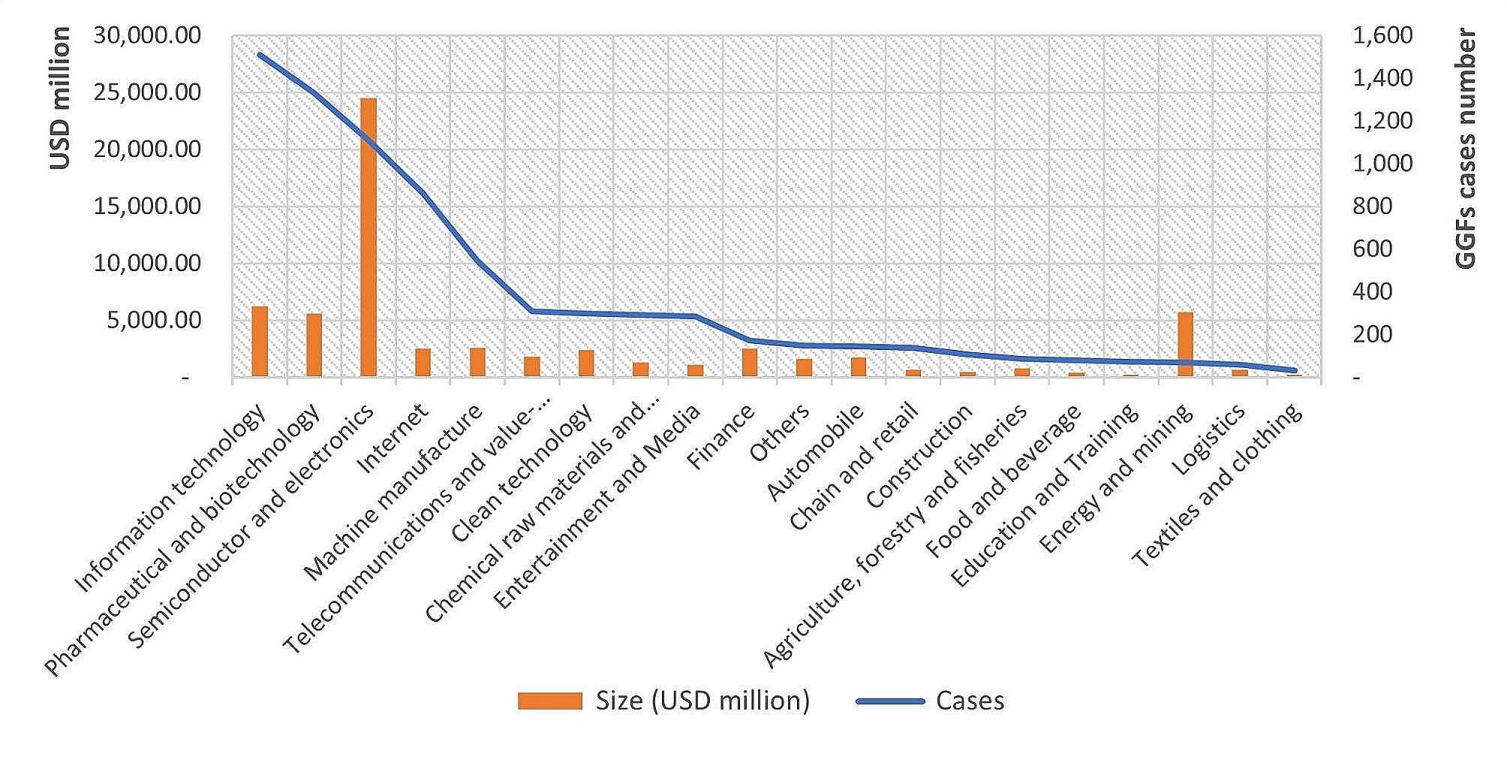
GGFs’ investments by cases and size in different sectors from 2000 to August 2021 Source: Authors’ analysis of data from PEDATA

**Fig. 3:**
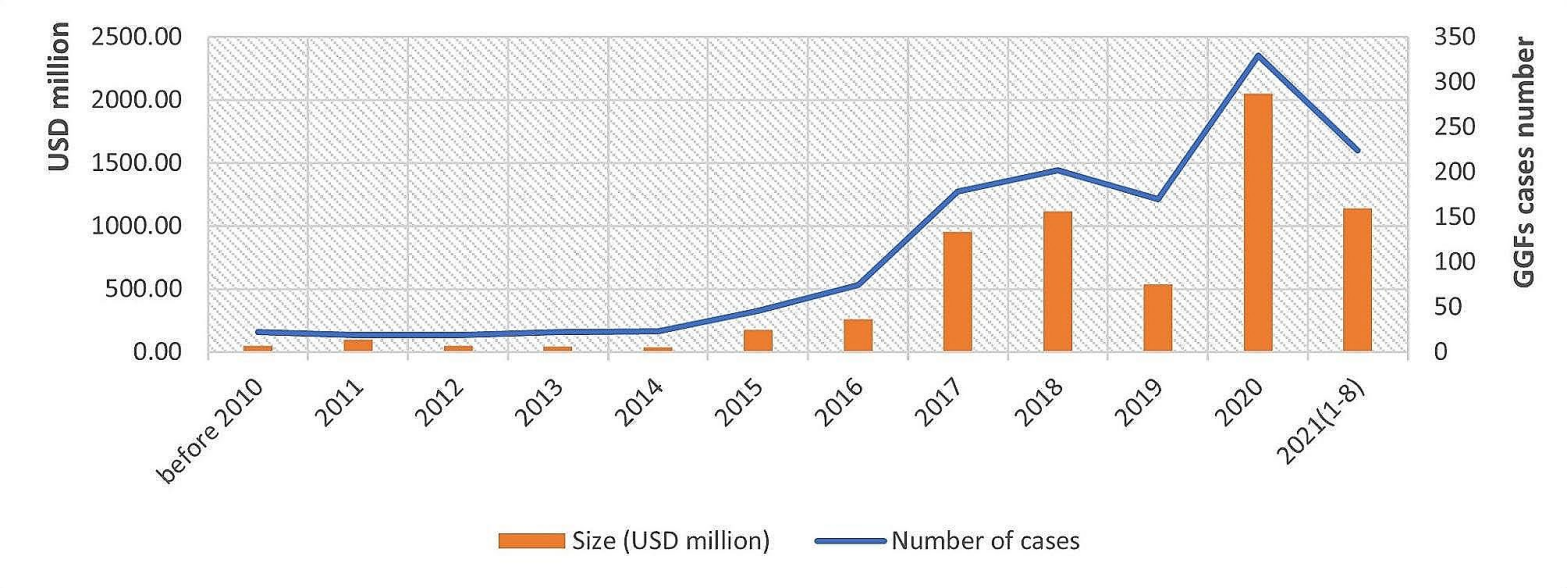
GGFs’ investments in P&B from 2000 to August 2021 Source: Authors’ analysis of data from PEDATA

GGFs have invested in a wide range of industries, of which the most significant ones are strategic emerging industries. Semiconductors, electronics, P&B, and energy receive the most investment from 2000 to August 2021 (Fig. 2). The semiconductor industry has received extremely significant GGF investment, linked to competition between China and the US from 2018 (see discussion below). As a strategic emerging industry in China, the P&B sector has also attracted significant investment. Fig. 3 shows 1,328 investments, placing P&B second by number of investment cases, with a disclosed investment over RMB 42.1 billion (about USD 6.5 billion), rating 4th in overall volumes of investment. GGFs’ activity in this sector began to rise in 2015, increased sharply in 2017, and peaked in 2020.

**Fig. 4:**
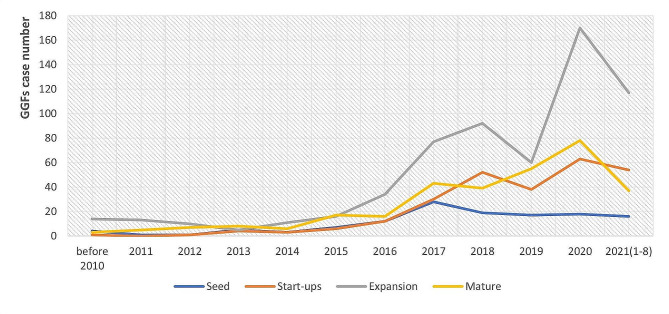
Investment stages of GGFs in P&B by case from 2000 to August 2021 Source: Authors’ analysis of data from PEDATA

Our data show that oncology and chronic disease therapies receive the most funding from GGFs among all new medicine pipelines in the P&B sector. However, potentially less commercially lucrative pipelines, such as antimicrobials and anti-infective drugs, and vaccines for communicable diseases, have attracted little funding. Only 28 investments have been made into such pipelines out of 420 disclosed investments in new medicine pipelines, or approximately 7%. This result is contrary to our expectations, given the stress in both national and local policies^2^ on the need to prioritise R&D with a social purpose and for new medicines that meet a social need. Our data, however, do not show GGFs prioritise such investments in their portfolios.

GGFs’ investments also differ by the stage of development of companies they invest in. Fig. 4 shows that GGFs’ investments are concentrated in companies in the expansion stage, with less invested in companies in early stages. Such investments have grown significantly more than investment in start-ups and mature companies, and seed-stage companies continue to be left behind.

#### How to understand GGFs investments in P&B?

After mapping investments in the P&B sector, we interviewed GGF executives and managers and explored how they understand investing in P&B. Three main themes emerged from the interviews and are discussed here: that GGFs investments in different sectors respond to government policy; that specific government priorities in P&B help determine the focus of investments; and that GGFs tend to be late-stage investors, limiting their support to innovation.

#### GGFs’ investments are responsive to changing government policy

**Fig. 5:**
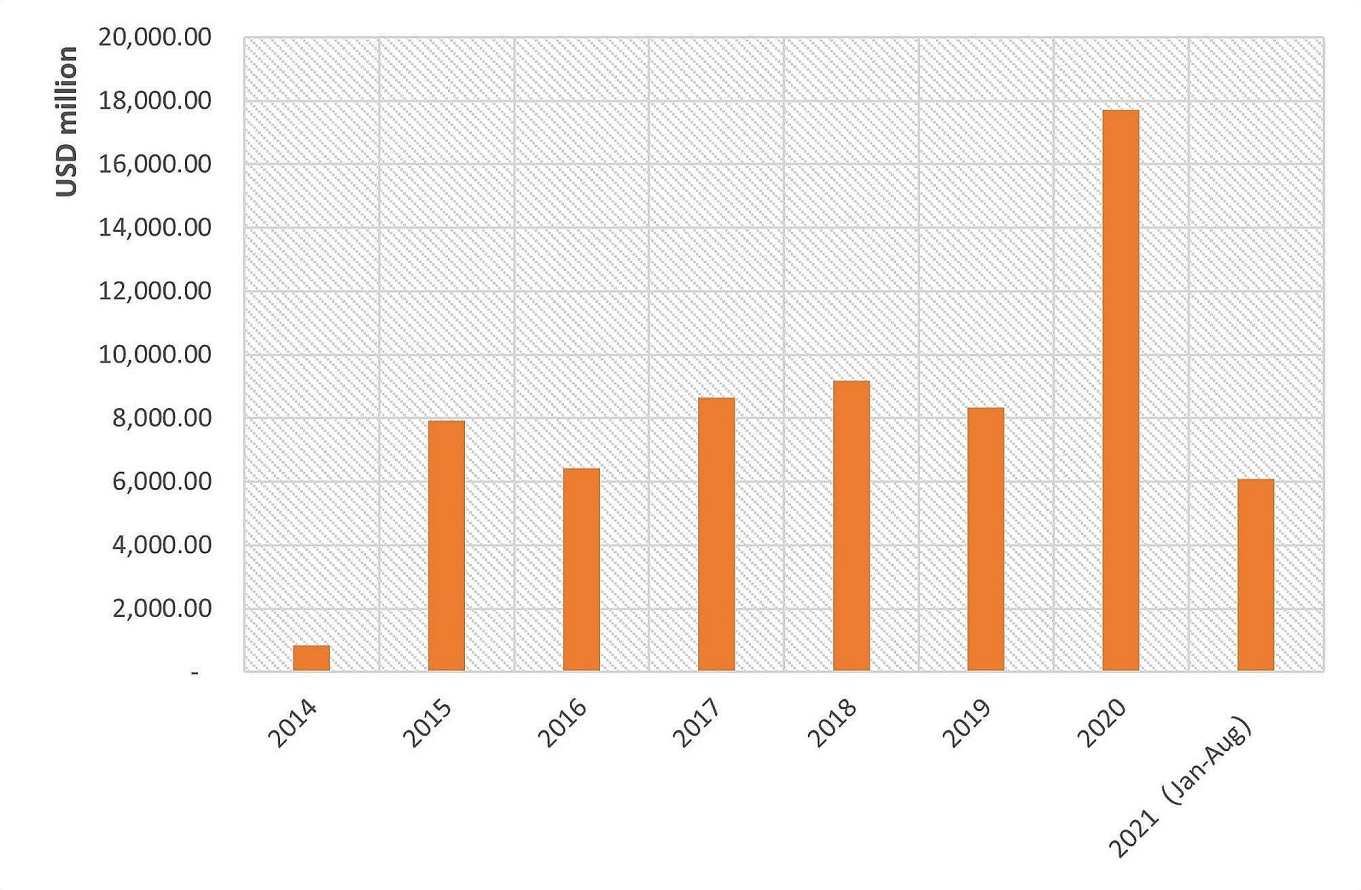
GGFs’ investments from 2014 to August 2021 (USD million) Source: Authors’ analysis of data from PEDATA

**Fig. 6:**
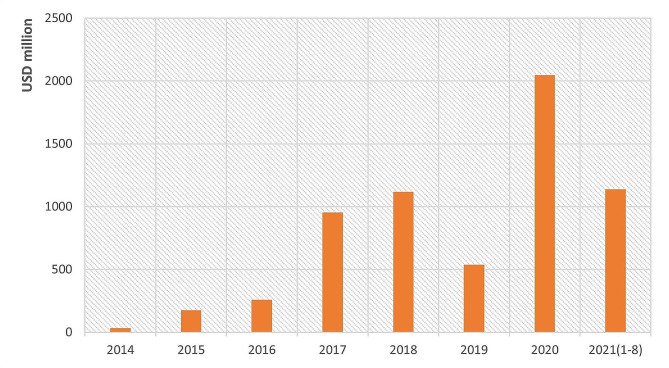
GGFs’ investments in P&B from 2014 to August 2021 Source: Authors’ analysis of data from PEDATA

Existing literature points to P&B investments in China being highly policy-sensitive [53], and our interviewees (D, F, Q) argue that changes in industrial policy priorities have been the key determinant of GGFs’ investments in P&B in the last decade. On the advice of our interviewees, we compared the overall trend in GGFs’ investments with the trend of their investments in P&B and find that they differ. While the number of GGFs increased in 2015 (Fig. 5), their investments in P&B did not change significantly. Instead, P&B saw dramatic growth in 2017 (Fig. 6), as GGFs, overall, started to plateau.

According to our interviewees, this reflects changes in government policy priorities for P&B, including the release of a national policy identifying P&B as a priority industry [56], and subsequent reforms, including dynamic updating of the National Reimbursement Drug List (NRDL), accelerated marketing authorisation of innovative drugs, and reforms to the hospital payment system.^3^ These coordinated reforms reflect a strategic, coordinated, and mission-driven approach to development of the sector. This has started to create new markets for pharmaceutical companies, especially innovative drug developers (for example, sales of Herceptin and Avastin have increased substantially following inclusion in the NRDL [57] and have directly stimulated investment in the pharmaceutical sector (interviewee Q; 58).

#### GGFs’ investments are in line with government’s priority sectors – and market demand

All interviewees acknowledge that industrial policies guide GGFs’ investments. A recent high-level policy on priorities for development of P&B in China [56] provides technical guidance on developing new drugs, vaccines, and essential drugs for which there is an unmet clinical need. National and local industrial policies prioritize new drug development in the areas of oncology, cardiovascular diseases, diabetes, neurodegenerative diseases, psychiatric diseases, highly prevalent immune diseases, major infectious diseases, and rare diseases [56]. In practice, sub-national governments introduce industrial policies based on local conditions and in line with national policy [58, 59].

GGFs’ perceptions of the priorities of pharmaceutical and biotechnological industrial policies lead them to mainly concentrate their investments in oncology and chronic disease drugs (interviewees B, D, F, P, R). Interviewee F explains the rationale for investing in oncology drugs: not only is there potentially high demand if the drug is successful, but such investments are likely to receive national and/or local government support (to clinical trials, registration, and commercialisation), and follow on funding is likely to be easier to get, should it be needed. This shows how policy and market demand are frequently linked, including in national P&B policies, which stress the importance of clinical need [56]. Moreover, although GGFs are not required to make great profit, executives and managers have a default understanding that GGFs should not *lose* money A, B, F and O), leading to GGFs favouring new drugs viewed as policy priorities, as having potential high-demand, and being potentially profitable.

When we probed interviewees as to why few investments are made in antibiotics, or other pharmaceuticals with a ‘public goods’ character, informants told us that they have not really considered this issue or rarely receive business plans for antibiotics or similar drugs. Counterintuitively, despite the national level, strategic, target to develop new antimicrobials, evidence from our interviews shows this is not viewed as a policy priority by GGFs.

#### GGFs’ risk aversion and limited talent pool skew them towards later stage investments

GGFs come in different kinds: start-up guidance funds, industrial guidance funds, and public-private partnership funds [60]. Of these three types, industrial guidance funds predominate. Such funds focus on industrial upgrading and development rather than supporting start-ups, which is likely to explain why there is limited early-stage investment. In P&B, this tendency reflects the risks of failure in the early stages of drug development, and the absence of ‘fault tolerance’ mechanisms, which limit GGFs’ appetite for risk (interviewees A, B, D, E, H, I, N, P). In P&B, most GGFs are therefore *de facto* ‘non-early stage’ funds (interviewee D), despite the policy intent behind them, which clearly includes support for innovative activities and early-stage investment, which is insufficiently provided by the private sector. Despite the evidence showing that GGFs can support innovation, therefore, there are likely to be limits to how this applies in P&B.

An insufficient talent pool is another constraint to GGFs supporting early-stage companies or programmes. Investing in P&B innovation requires professional staff with both an industrial background and knowledge of investment practice. However, competent professionals usually work for top international investors, rather than for GGFs (Interviewee B), and most GGF staff lack medical or biotechnology knowledge (interviewees B, C, F, Q, R). This appears to be a critical issue affecting the role GGFs can play in supporting innovation in P&B (see Sect. 4.2).

#### Summary

Our interviews show that while industrial policies play a vital role in GGFs’ decision-making in P&B, the drivers of GGFs behaviour are nuanced. They do not invest solely based on policy signals, but also assess the extent to which there will be a market for a given drug, whether because it aligns with China’s disease burden and public demand, or because of ‘pull incentives’ created through adjustments to government purchasing and reimbursement lists. In addition, our interviews show that there is, at best, a very weak policy signal coming through regarding the need for investment in companies developing antibiotics or bringing them to market. There are also concerns about GGFs’ capacity to really play the role as an early-stage, risk-taking investor, given their capacity constraints and absence of fault tolerance mechanisms. This, and GGFs’ having to balance an industrial development mandate with a profit motive, highlights a contradiction in how GGFs operate and their potential contribution in P&B.

### GGFS’ potential to contribute to mission-driven innovation in P&B

The last section shows that GGFs face a variety of structural constraints in supporting innovation in P&B, including for products where there is a limited market – such as antimicrobials – even if these are a policy priority or of social importance. In our interviews, we wanted to more fully explore GGFs’ potential role in supporting ‘mission-driven’ R&D in P&B, given China’s willingness to use a range of industrial policy tools to support innovation and strategic industrial development, its increasing importance in P&B, and the government’s stated desire for China to play a larger role in supplying global public goods for health.

Our interviews therefore explored elements of mission-driven innovation and the extent to which GGFs align with, or support, the functions of this. This section discusses findings in three areas we probed with interviewees: whether GGFs should be understood as having a ‘social’ mandate in the way they approach investment decisions; their role in setting the direction of industrial development; and their role in creating and shaping markets. The section concludes with a discussion of some limitations of GGFs in supporting mission-driven innovation.

#### How should we interpret the social function of GGFs?

The concept of mission-driven innovation includes the requirement that government help set the direction of development in line with social goals. To better understand how this function is operationalised through GGFs, we reviewed policy and literature on the purpose and functioning of GGFs, and questioned our interviewees regarding their understanding of the mandate of GGFs in promoting social goals, including – as in our study – pharmaceuticals such as antimicrobials that have characteristics of a ‘public good’ or ‘merit good’.

Our review is inconclusive. Policies frequently state the requirement that GGFs address systemic bottlenecks to industrialisation and development, but this requirement is unspecific. Meanwhile, our interviews show a lack of clarity over GGFs’ social mandate, that different groups may understand this differently, but that policy changes may be promoting an increase in the social component of GGFs’ mandate:

- A number of GGF executives closely connected to government argue that GGFs should, *de facto*, be considered an extension of government because they are government-funded and -mandated (interviewees E, H, O, P and R), and that this gives them a composite responsibility, of promoting economic development *and* creating social benefit. They state that policies are being adjusted to give GGFs a more explicitly social mandate: a recent Ministry of Finance policy [61], for example, calls for GGFs to focus on ‘key and innovative sectors that require government intervention’ and ‘areas in urgent need of economic *and social* development’ [emphasis added]. This is leading some GGFs to change their operations, though GGFs still need to ensure profitable operation to be sustainable (interviewee P).
- The views of managers, responsible for making operational decisions, differ from the first group, viewing GGFs as having only an indirect social mandate. These managers predominantly state that government should support social goals through taxation and subsidies, and that GGFs can contribute through promoting growth, tax revenues, and improving the government’s capacity to invest in such social goals (interviewees C, D, F, J, K and M).

#### How do GGFs articulate ‘directionality’ to help achieve social priorities?

‘Directionality’ is a fundamental feature of mission-driven approaches to innovation, through which social purpose is articulated and translated into actions that can direct innovation towards that purpose. In this paper, novel antimicrobials are an example of an under-supplied pharmaceutical, which nevertheless serves an important social function. The interviews indicate two main ways in which GGFs can contribute to establishing the direction of innovation, though neither is fully exploited in the way GGFs currently function.

- Setting GGFs’ investment focus. All interviewees confirmed that the government decides the investment focus of a GGF when it is established, and that other actors have little influence in this. This shapes subsequent investment behaviour and is therefore the most significant moment at which government influences a fund’s behaviour. For example, the scope of a fund could be defined as ‘advanced pharmaceutical and biotechnology technologies’ or, in more detail, as focusing on a specific disease or category of disease, such as ‘new oncology drugs’.
- Setting GGFs’ investment stage. As discussed above, the stage at which a fund invests influences the extent to which it can support innovative technologies, and GGFs tend to make late-stage investments. In the P&B sector, a mature company is viewed as one that has completed drug discovery, received approvals to start clinical trials, or even entered Phase II trials (interviewees B, F, P, Q and R). Interviewees state that GGFs’ investment in P&B is changing rapidly, following COVID and changing government guidance (interviewees F, Q, R).^4^

In other words, government has levers through which it is able to influence GGFs’ behaviour, and GGFs’ responsiveness to policy indicates that changing policies have the potential to change their investment focus. The change in focus following COVID-19 is to be expected, as government increasingly realises the strategic and social importance of P&B.

#### Market creation and shaping

Market creation and shaping is a core part of mission-driven approaches to innovation, reflecting the ‘joined up thinking’ that such approaches call for. An example is the use of purchasing mechanisms to stimulate the development of innovative pharmaceuticals (as in China’s NRDL), or to compensate for insufficient market demand in the case of antibiotics. While GGFs do not directly shape markets, they can be responsive to market-shaping efforts.

- GGFs’ understanding of market potential as a driver. Our interviewees did not explicitly distinguish between drugs with a public goods character and for which there is insufficient demand, such as antibiotics, and others. On the contrary, they argued that potential market size is key to whether GGFs would invest in a given innovative drug (interviewee F, L, P and Q), as well as a drug’s clinical effectiveness, whether first-in-class or me-too (interviewee F and Q).
- Probing antibiotics more specifically, interviewees argued that China’s large population base may provide a sufficient market even for drugs such as antibiotics (interviewee L and Q), whose use must be carefully stewarded, especially if they were included the NRDL (interviewees B, D, J, K, L, P and Q). This is echoed by a report from one innovative Chinese antibiotic developer [62]. This is a curious finding, but is likely related to an insufficient understanding of the need for stewardship of novel antimicrobials.
- Strong industrial policies. In addition to the discussion above of the importance of policy direction in P&B, almost all our interviewees mentioned changes in the semiconductor sector to underline the importance of policy in shaping GGFs’ behaviour and aligning it with national priorities. Prior to the US ban on technology exports to China, investments in this sector were limited.^5^ However, starting with the US-China dispute, Chinese industrial policy was rapidly adjusted, and investment by government and GGFs rose rapidly and dramatically, with the aim of substantially increasing China’s share of global chip manufacturing, complemented by the likelihood of growth in domestic demand for Chinese-produced chips.^6^

#### Factors limiting GGFs contribution to social goals and public goods in P&B

Our interviews explored factors that may impede GGFs from playing a larger social role and contributing to the development of pharmaceuticals with a public goods character, such as antimicrobials. This section synthesises the main factors stressed by our interviewees.

- Insufficient talent pool. Section 3 notes that a limited talent pool is a constraint to GGFs’ investing effectively in P&B. Funds struggle to offer competitive income to attract qualified and competent staff (interviewees B, D, F, G, H, J, O, P, Q). The development of novel antimicrobials illustrates this – this requires highly specialised knowledge, especially given trends towards use of artificial intelligence (AI) in new antibiotic R&D [63, 64]. GGFs face great challenges hiring staff able to effectively assess such technologies and companies.
- Insufficient fault-tolerance mechanisms. As discussed above, fault tolerance mechanisms reduce GGFs’ willingness to invest in risky, but potentially important, technologies and companies. This is despite high-level government policy stating the need for such mechanisms [65].
- A third constraint is the absence of demand-creation mechanisms that could complement the supply-side support that GGFs are, in theory, able to provide to the development of public goods such as novel antimicrobials. In the case of antimicrobials, such mechanisms are very new, and currently only being trialled in a handful of cases [10].

## Discussion: What role for GGFS in China’s mission-driven P&B innovation and provision of public goods?

Mission-driven innovation for social ends requires clear aims and joined-up policy support. This includes supporting scientific research in line with public needs, but also support to companies to commercialise discoveries, appropriate regulation, and demand stimulation or market creation to provide an outlet for products. This study has shown GGFs to have a hybrid identity, of being guided by policy – which can articulate strategic, social and developmental goals – but also constrained by market forces. Most lack a clear mandate to pursue specific goals and do not specifically consider social aims in their investment decisions.

First, the social role of GGFs, and the way ‘directionality’ influences their operations, are unclear. While Chinese government policy towards P&B is strategic, aiming to promote innovation and market upgrading, and our interviews show that GGFs are responsive to policy, the way this is operationalised is diffuse, rather than targeted at specific priority technologies. This, along with the fact that government generally plays a limited role in GGFs’ operations once a fund has been set up, limits its ability to directly influence priorities. GGF staff are divided over whether GGFs have a mandate to include social considerations such as public benefit in their decision making, and this is at best a weak factor informing their investments.

Second, GGFs are, effectively, constrained within a market logic. The requirement to produce a return on investment, the absence of specific government financing conditions or flexibilities (e.g. subsidies, discount rates) for projects with a clear social benefit or public goods character, and limited fault tolerance, make them function in a similar way to commercial funds, diluting any explicitly social mandate they might have.

Third, there are limited mechanisms to support demand for public goods-type products, reflecting an absence of the kind of joined up policy required for mission-driven innovation. Mechanisms such as the NRDL can play a role in overall sectoral upgrading, but based on our research, are unable to target products with limited markets, such as antimicrobials. They differ from mechanisms such as NICE’s subscription model, whose innovation is to separate the need for a product and volumes of product used [10]. To date, there has been no experimentation with this kind of focused pull mechanism for antimicrobials in China, indicating the limited linkage between the health ministry and GGFs, at least as regards antimicrobials. Equally, in areas such as semiconductors, where GGFs have been highly responsive, their response has been underpinned by increasing market demand linked to US-China trade frictions. In the absence of innovative pull mechanisms in China of the kind being trialled elsewhere [2, 66, 67], GGFs are highly unlikely to invest in antimicrobials or other products for which there is a need, but limited market.

Fourth, a number of contextual factors reduce the extent to which GGFs currently demonstrate an ability to support mission-driven innovation. First is the context in which they were introduced – one in which government’s priority was industrial catch up in key sectors, rather than solving grand challenges, though there is some evidence that social considerations are increasingly important in (some) GGFs’ functioning. Second, is GGFs’ limited capacity to prioritise investments, related to China’s current stage of development and their challenges in attracting certain kinds of talent. Third is the newness of GGFs – the majority were founded after 2016, meaning that they have not yet undergone a full project cycle. Given this, it is likely that we will see adjustments in their functioning over time.

Fifth is a contextual factor related to China’s current stage of development. As noted above, much Chinese P&B strategy revolves around catch up. This points to a conundrum in theories of mission-driven innovation – China’s P&B sector is starting from a relatively low base, as China has come out of the planned economy period and started to deliberately develop the sector. Until recently, much of the thrust in government policy towards the sector has been geared towards provision of basic medicines, volume exports, and increasing self-sufficiency [68]. In antimicrobials, China’s historic mission has largely been to ensure self-sufficiency, reflecting the fact that the country’s antibiotic industry was originally developed at a time of western scientific blockade [69]. Despite the country’s substantial (and increasing) scientific capacity, there may be rational reasons for government to assess that the country’s current priorities should be elsewhere.

Finally, the examination of this Chinese case points to a difference in framing between Mazzucato’s mission-driven innovation and Chinese approaches, related to recent history. Theories of mission-driven innovation have primarily been developed with reference to developed, western market economies. They come at a time when scholars are calling for more active industrial policy in these countries amid the dominance of market theories and a decline in the use of industrial policy in the latter part of the twentieth century [70]. However, this context is very different from that of China, where government has never retreated from economic activity, despite the increasingly important role of the market over the last forty years. In other words, theories of mission-driven innovation have a relatively clear logic in the context of highly marketized western economies, but are not necessarily so powerful in understanding the drivers of China’s recent economic and industrial development, though – as with the case of semiconductors discussed above – this may now be changing.

## Conclusions

The article has discussed the role of GGFs in investing in P&B and explored their potential contribution to mission-driven innovation. It shows them to have a limited role in supporting the development of antimicrobials – a category of pharmaceutical with a strong ‘public goods’ character – based on evidence from investment trends and interviews with GGF staff. Furthermore, contrary to our expectations, the study finds that GGFs do not have a clear social mandate, despite their status as strategic investment vehicles supporting China’s development.

One important finding is that GGFs are highly sensitive to policy changes. It is therefore possible that we will see changes in their operation, given the Chinese government’s changing priorities for P&B as a sector, and in the context of tensions between China and many western countries and of China’s increasing promotion of self-reliance in key areas of the economy [71, 72].

However, our analysis also indicates that there are a number of challenges to GGFs playing a more effective role. We find that GGFs are constrained by a market logic that, without compensating mechanisms, limits their investment in areas with limited or uncertain financial returns, whatever the potential social benefit. There are also practical constraints to GGFs’ operation, including the limited role for government in guiding the focus of investment, limited human resources, and a lack of linking between GGFs (as a supply-side mechanism) and demand-side mechanisms.

This article has presented a preliminary exploration of GGFs, but there is potential for more research in this area, given the breadth of areas in which GGFs invest and their potential significance in the Chinese.

economy. GGFs remain a very new investment vehicle in China, and how they evolve in response to the changing needs of the Chinese economy will be important to assess, as will the changing demands on them from Chinese government and policymakers, and the institutional reforms and strengthening needed to allow them to play a more effective role. Given GGFs scale and strategic importance, they deserve further research as China’s P&B sector becomes increasingly globally important, and as the Chinese government commits to a larger role in global health.

### Electronic supplementary material

Below is the link to the electronic supplementary material.

Supplementary Material 1

Supplementary Material 2

## Data Availability

Data sourced from PEDATA on Chinese GGFs operations in the P&B sector were compiled in Excel and are available from the corresponding author on request. Interviewees were guaranteed anonymity, meaning that original data from the interviews is not being made publicly available.

## Footnotes

1 Data were accessed via the PEDATA website (www.pedata.cn) in autumn 2021. The website is at present only accessible from inside China.

2 Supporting policies exist at both national and local levels. The National Action Plan (2016–2020) on the containment of antimicrobial resistance (link) is an example of national level policies, whereas Shanghai government issues ongoing policies [59], for example.

3 The National Healthcare Security Administration (NHSA) has issued a dynamic adjustment mechanism for the NRDL in 2018, reducing the NRDL’s adjustment time to a year basis. Prior to 2018, the catalogue had only been adjusted in 2000, 2004, 2009, 2015 and 2017. Details can be visited from NHSA’s website.

4 Such early-stage GGFs are not common. However, examples include the establishment of an early-stage biomedical GGF in 2021 (link), driven by government, focusing on innovative drugs from the discovery stage. A similar attempt was made in Shenzhen, where a GGF was set up as an angel investor in 2018 (link), focusing on ‘critical, strategic and cutting-edge’ programmes, and with CNY 10 billion (around USD 1.54 billion) under management.

5 The US-China tech conflict, focusing on core high-tech technologies such as 5G, artificial intelligence (AI) and semiconductors, has continued from the Trump administration to the current Biden administration. The US high-tech embargos on China began with the imposition of high-tech technology and product export controls on China in 2018 and have gradually expanded to comprehensive bans on investment, people exchange, and research and development. Semiconductors are one of the sectors that have been hit the hardest, and the US sanctions have forced China’s semiconductor industry into a local research and production substitution process.

6 Deloitte’s statistics (2021) illustrate these investments’ changes in 2018 and 2019, where China’s government investments in semiconductors soared from 0.95 million to 31.1 billion dollars, more than 30 times in size. This change in size is supposed to affect chip manufacturing capacity, of which China’s capacity is projected to increase from 21 to 45% globally. [see Deloitte, Anchor of Global Semiconductor, Asia Pacific Takes Off, accessed on 14 Nov 2021 at: https://www2.deloitte.com/cn/en/pages/technology-media-and-telecommunications/articles/tmt-anchor-of-global-semiconductor-industry-2021.html. ]

## Abbreviations

AMR: Antimicrobial resistance
GGF: Government guidance fund
NHSA: National Healthcare Security Administration
NRDL: National Reimbursement Drug List (
P&B: Pharmaceuticals and biotechnology
R&D: Research and development
S&T: Science and technology
VC: Venture capital
