# Supplementary material for: Towards mission-driven investment in new antimicrobials? What role for Chinese strategic industrial financing vehicles in responding to the challenge of antimicrobial resistance?
Source: Global Health. 2024 Mar 26;20:26. doi: 10.1186/s12992-024-01030-2 (PMC10967126; doi:10.1186/s12992-024-01030-2)
Supplement: Supplementary file 1 — Supplementary Material 1 [file 12992_2024_1030_MOESM1_ESM.pdf]

Additional file 1: List of interviewees

Interviewee	Position	GGF's Location	Investing in P&B	Interview date
A	Managing director	Henan province	Yes	16 Oct 2021
B	Investment director	Shaoxing City and Beijing	Yes	17 Oct 2021
C	Director	Beijing		18 Oct 2021
D	Investment director	Shanghai	Yes	18 Oct 2021
E	Chief person	Gansu Province		20 Oct 2021
F	Partner	Shanghai	Yes	21 Oct 2021
G	Investment director	Beijing		22 Oct 2021
H	Chief person	Beijing		22 Oct 2021
I	Investment manager	Anhui Province		23 Oct 2021
J	Board director	Sichuan Province		23 Oct 2021
K	Investment manager	Beijing		25 Oct 2021
L	Investment manager	Beijing	Yes	25 Oct 2021
M	Investment manager	Henan province		26 Oct 2021
N	Investment manager	Shandong Province		27 Oct 2021
O	Vice president	Henan province		28 Oct 2021
P	Board director	Sichuan province	Yes	29 Oct 2021
Q	Managing director	Shenzhen	Yes	03 Nov 2021
R	Vice president	Shenzhen	Yes	11 Nov 2021
