# Supplementary material for: Towards mission-driven investment in new antimicrobials? What role for Chinese strategic industrial financing vehicles in responding to the challenge of antimicrobial resistance?
Source: Global Health. 2024 Mar 26;20:26. doi: 10.1186/s12992-024-01030-2 (PMC10967126; doi:10.1186/s12992-024-01030-2)
Supplement: Supplementary file 2 — Supplementary Material 2 [file 12992_2024_1030_MOESM2_ESM.pdf]

Additional file 2, GGFS policy timeline

Date	Policies
2002.06.29	Law of the PRC on the Promotion of Small and Medium-sized Enterprises<br>[In Chinese: 中小企业促进法]
2005.11.15	Interim Measures for the Administration of Start-up Investment Enterprises<br>[In Chinese: 创业投资企业管理暂行办法]
2006.02.09	National Medium-and-long-term Program for Scientific and Technological Development (2006-2020)<br>[In Chinese: 国家中长期科学和技术发展规划纲要]
2007.07.06	Interim Measures for the Administration of the Venture Capital Guiding Fund of Small and Medium-Sized Technological Enterprises<br>[In Chinese: 科技型中小企业创业投资引导基金管理暂行办法]
2008.10.18	Guiding Opinion of regulating the formation and operation of the Venture Capital Guiding Fund<br>[In Chinese: 关于创业投资引导基金规范设立与运作的指导意见]
2010.12.09	Interim Measure for the collection of Equity Investment Income of the Venture Capital Guiding Fund of Small and Medium-Sized Technological Enterprises<br>科技型中小企业<br>[In Chinese: 科技型中小企业创业投资引导基金股权投资收入收缴暂行办法]
2011.07.07	Interim Measures for the Management of the National Guiding Fund for the Conversion of Scientific and Technological Achievements<br>[In Chinese: 国家科技成果转化引导基金管理暂行办法]

2014.05.13	Notice of the General Office of the National Development and Reform Commission on Further Effectively Conducting the Relevant Work of Supporting the Development of Venture Capital Enterprises<br>[In Chinese: 国家发展和改革委员会办公厅关于进一步做好支持创业投资企业发展相关工作的通知]
2014.05.21	State Council Executive Meeting<br>[In Chinese: 国务院常务会议]
2014.08.08	Interim Measures for the Administration of the establishment of venture capital sub-funds by the National Guiding Fund for the Conversion of Scientific and Technological Achievements<br>[In Chinese: 国家科技成果转化引导基金设立创业投资子基金管理暂行办法]
2014.11.16	Guiding Opinions of the State Council on Innovation of Investment and Financing Mechanisms in Key Fields to Encourage Social Investment<br>[In Chinese: 国务院关于创新重点领域投融资机制，鼓励社会投资的指导意见]
2015.01.14	State Council Executive Meeting<br>[In Chinese: 国务院常务会议]
2015.09.01	State Council Executive Meeting<br>[In Chinese: 国务院常务会议]
2015.11.12	Interim Measures for the Administration of Government Investment Funds<br>[In Chinese: 政府投资基金暂行管理办法]
**Date**	**Policies**
2015.12.04	Interim Measures for the Administration of the National Guiding Fund for the Conversion of Scientific and Technological Loan Risk Compensation

	[In Chinese:]国家科技成果转化引导基金贷款风险补偿管理暂行办法]
2015.12.25	Guiding Opinions of the Ministry of Finance on Injecting Financial Funds into Government Investment Funds to Support Industry Development<br>[In Chinese: 财政部关于财政资金注资政府投资基金支持产业发展的指导意见]
2016.07.18	Opinions of the Central Committee of the Communist Party of China and the State Council on Deepening the Reform of the Investment and Financing System<br>[In Chinese: 中共中央、国务院关于深化投融资体制改革的意见]
2016.09.20	Several Opinions of the State Council on Promoting the Sustainable and Sound Development of Venture Capital<br>[In Chinese: 国务院关于促进创业投资持续健康发展的若干意见]
2016.12.30	Interim Measures for the Administration of Government-Sponsored Industry Investment Funds<br>[In Chinese: 政府出资产业投资基金管理暂行办法]
2017.04.27	Plan of the State-owned Assets Supervision and Administration Commission of the State Council for Promoting the Transformation of Functions with the Focus Put on Capital Management<br>[I Chinese: 国务院国资委以管资本为主推进职能转变方案]
2019.05.05	Regulation on Government Investment<br>[In Chinese: 政府投资条例]
2020.02.24	Notice by the Ministry of Finance of Strengthening the Management of Government Investment Funds and Improving the Benefits of Fiscal Contributions<br>[In Chinese: 财政部关于加强政府投资基金管理提高财政出资效益的通知]

Source: The PRC State Council, Ministry of Finance, National Development and Reform Commission
